# P-427. Prevalence of Healthcare-Associated Infections: 2023 Point Prevalence Survey in 218 U.S. Acute Care Hospitals

**DOI:** 10.1093/ofid/ofae631.628

**Published:** 2025-01-29

**Authors:** Nora Chea, Taniece R Eure, Rebecca Alkis Ramirez, Joelle Nadle, Jane E Lee, Monica Lehmann, Lyndzie Sardenga, Christopher A Czaja, Helen Johnston, Melissa Kellogg, Catherine E Emanuel, Alana Cilwick, Maria Correa, Meghan Maloney, Susan M Ray, Jessica R Howard-Anderson, Stacy Carswell, Rebecca Perlmutter, Kaytlynn Marceaux-Galli, J P Mahoehney, Ruth Lynfield, Marla Sievers, Cory Cline, Melissa Judson, Donita C Gorman, Ghinwa Dumyati, Christine Hurley, Elizabeth Keller, Marissa Walsh, Erin Licherdell, Julia Tellerman, Christina B Felsen, Rebecca Pierce, Valerie Leigh S Ocampo, Monika E Samper, Kimberly A Hires, Lauren T Adrian, Roza Tammer, Alexia Y Zhang, Shannon Hiratzka, Angela Dusko, Christopher Wilson, Melphine Harriott, Daniel Muleta, Casey L Morrell, Darryl C Nevels, Cullen Adre, Shelley Magill, Cheri Grigg

**Affiliations:** Centers for Disease Control and Prevention, Atlanta, Georgia; Centers for Disease Control and Prevention, Atlanta, Georgia; CDC, Atlanta, Georgia; California Emerging Infections Program, Oakland, California; California Emerging Infections Program, Oakland, California; California Emerging Infections Program, Oakland, California; California Emerging Infections Program (CEIP), San Diego, California; Colorado Department of Public Health and Environment, Denver, Colorado; Colorado Department of Public Health, Denver, Colorado; Colorado Department of Public Health and Environment, Denver, Colorado; Colorado Department of Public Health & Environment, Parker, Colorado; Colorado Department of Public Health and Environment, Denver, Colorado; Connecticut Emerging Infections Program, Yale School of Public Health, New Haven, Connecticut; Connecticut Department of Public Health, Hartford, Connecticut; Emory University School of Medicine, Atlanta, Georgia; Emory University, Atlanta, Georgia; Emory University, Atlanta, Georgia; Maryland Department of Health, Baltimore, Maryland; Maryland Department of Health, Baltimore, Maryland; Minnesota Department of Health, Saint Paul, Minnesota; Minnesota Department of Health, Saint Paul, Minnesota; New Mexico Department of Health Epidemiology and Response Division, Santa Fe, New Mexico; New Mexico Department of Health, Albuquerque, New Mexico; New Mexico Department of Health, Albuquerque, New Mexico; NMDOH, Santa Fe, New Mexico; New York Emerging Infections Program and University of Rochester Medical Center, Rochester, New York; University of Rochester, Center for Community Health and Prevention, Rochester, New York; University of Rochester Medical Center, Rochester, New York; New York Emerging Infections Program, Rochester, New York; University of Rochester Medical Center, Rochester, New York; New York Emerging Infections Program, Rochester, New York; University of Rochester, Rochester, NY; Centers for Disease Control and Prevention, Atlanta, Georgia; Oregon Health Authority, Portland, Oregon; Oregon Health Authority, Portland, Oregon; Division of Healthcare Quality Promotion, National Center for Emerging and Zoonotic Infectious Diseases, Centers for Disease Control and Prevention, Atlanta, Georgia; Oregon Health Authority, Portland, Oregon; Oregon Health Authority, Portland, Oregon; Oregon Health Authority, Portland, Oregon; Oregon Health Authority, Portland, Oregon; Oregon Health Authority, Portland, Oregon; Tennessee Department of Health, Nashville, Tennessee; TN Department of Health, Nashville, Tennessee; Tennessee Department of Health, Nashville TN, Antioch, Tennessee; Tennessee Department of Health, Nashville, Tennessee; Tennessee Department of Health - - Nashville, TN, Nashville, Tennessee; Tennessee Department of Health, Nashville, Tennessee; Centers for Disease Control and Prevention, Atlanta, Georgia; Division of Healthcare Quality Promotion, Centers for Disease Control and Prevention, Atlanta, Georgia

## Abstract

**Background:**

The Centers for Disease Control and Prevention (CDC) conducted point prevalence surveys in U.S. acute care hospitals in 2011 and 2015 to determine the prevalence and distribution of healthcare-associated infections (HAIs) and complement data reported to the CDC’s National Healthcare Safety Network (NHSN). HAI prevalence in hospitals was 4.0% in 2011 and 3.2% in 2015. We repeated the survey in 2023 to reassess hospital HAI prevalence and distribution. Here, we present the results of our preliminary analysis of the 2023 survey data.

**Figure:**
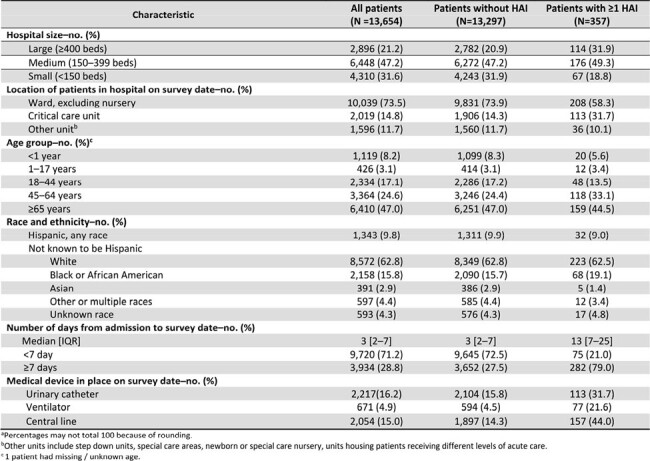

**Methods:**

Emerging Infections Program (EIP) sites in 10 states recruited up to 25 hospitals each, prioritizing hospitals that participated in the 2011 or 2015 survey. Each hospital selected a survey day between May 1 and September 30, 2023. Trained EIP staff reviewed medical records of patients randomly selected from the inpatient census on the survey day. Data collected included patient demographics, clinical characteristics, medical devices, hospital locations, and HAIs based on 2023 NHSN definitions. We described the characteristics of patients included in the survey and the percentage with HAIs.

**Figure:**
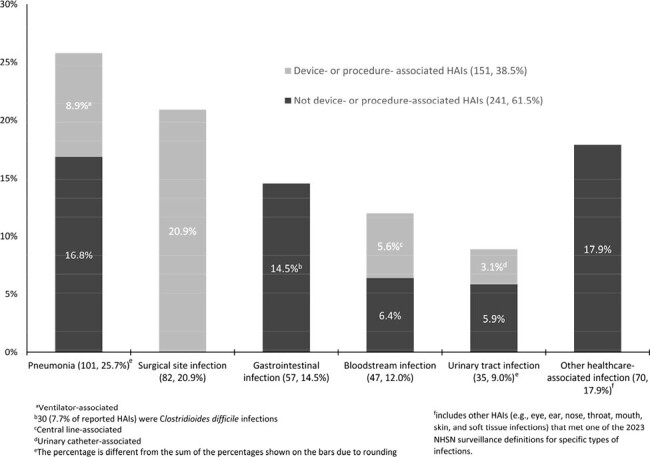

**Results:**

Of 13,654 patients in 218 hospitals, 357 (2.6%) had 392 HAIs. Compared to patients without an HAI, patients with HAIs were more frequently located in large hospitals, in critical care units, hospitalized ≥7 days on the survey date, and were more likely to have a central line, urinary catheter, or be on a ventilator on the survey date (Table). Among the 392 HAIs, 101 (25.8%) were pneumonia, 82 (20.9%) surgical site infection, 57 (14.5%) gastrointestinal infection, 47 (12.0%) bloodstream infection, 35 (8.9%) urinary tract infection, and 70 (17.9%) other HAI types. Most HAIs (241, 61.5%) were not associated with devices or procedures (Figure).

**Conclusion:**

HAI prevalence in acute care hospitals in 2023 was low. Almost two-thirds of HAIs were not associated with devices or procedures. These findings emphasize the need for continued infection prevention and control efforts in hospitals for all types of HAIs including those not associated with devices or procedures.

**Disclosures:**

**Meghan Maloney, MPH**, Pfizer Global R&D: Former employee, separated in 2009. I do have a retirement entitlement which I do not actively manage/no stock options. PGRD offers periodic buy outs

